# Co-existence of chlorosis inducing strain of Cucumber mosaic virus with tospoviruses on hot pepper (*Capsicum annuum*) in India

**DOI:** 10.1038/s41598-021-88282-9

**Published:** 2021-04-22

**Authors:** J. Vinodhini, L. Rajendran, R. Abirami, G. Karthikeyan

**Affiliations:** Department of Plant Pathology, Tamil Nadu Agricultural University, Coimbatore, 641 003 India

**Keywords:** Microbiology, Plant sciences

## Abstract

Cucumo- and tospoviruses are the most destructive viruses infecting hot pepper (chilli). A diagnostic survey was conducted to assess the prevalence of cucumo and tospoviruses in chilli growing tracts of Tamil Nadu. Infected plants showing mosaic with chlorotic and necrotic rings, veinal necrosis, mosaic mottling, leaf filiformity and malformation were collected. Molecular indexing carried out through reverse transcription polymerase chain reaction (RT-PCR) with coat protein gene specific primer of Cucumber mosaic virus (CMV) and tospovirus degenerate primer corresponding to the L segment (RdRp). Ostensibly, amplifications were observed for both CMV and tospoviruses as sole as well for mixed infections. The sequence analysis indicated that the Capsicum chlorosis virus (CaCV) and Groundnut bud necrosis virus (GBNV) to be involved with CMV in causing combined infections. The co-infection of CMV with CaCV was detected in 10.41% of the symptomatic plant samples and combined infection of CMV with GBNV was recorded in around 6.25% of the symptomatic plants surveyed. The amino acid substitution of Ser^129^ over conserved Pro^129^ in coat protein of CMV implies that CMV strain involved in mixed infection as chlorosis inducing strain. Further, the electron microscopy of symptomatic plant samples explicated the presence of isometric particles of CMV and quasi spherical particles of tospoviruses. This is the first molecular evidence for the natural co-existence of chlorosis inducing CMV strain with CaCV and GBNV on hot pepper in India.

## Introduction

The hot pepper commonly known as chilli (*Capsicum annuum* L.) is the most important spice crop with significant commercial value throughout the world due to its great phenotypic, horticultural, agricultural and biological diversity^1^. India is one of the world’s largest producer, consumer and exporter of chillies globally. Chilli is reported to be affected by more than 30 economically important viruses by causing 15–50% of yield losses in chilli cultivation^2,3^. It includes Cucumber mosaic virus^4^, Chilli leaf curl virus^5^, Tomato spotted wilt virus^6^, Groundnut bud necrosis virus^7^, Capsicum chlorosis virus^8^, Tobacco mosaic virus^9^, Potato virus Y^6^, Tomato leaf curl New Delhi virus^10^ and Chilli veinal mottle virus^11^. Among them the cucumo and tospoviruses are the most predominant viruses reported to infect almost all the vegetables crops and pose serious threat to chilli production^12^. CMV (genus *Cucumovirus*; family *Bromoviridae*) consist of isometric particles having sense, ssRNA genome with tripartite segment designated as RNA1, RNA2 and RNA3^13^. CMV causes severe mosaic symptoms like leaf distortion and fruit lesions which affect the significant marketable yield of chilli^14^. Tospoviruses (genus *Orthotospovirus*; family *Bunyaviridae*) are also isometric, ambi-sense, ssRNA virus with tripartite genome of small (S), medium (M) and large (L) segment^15^. The symptoms appeared on young leaves as chlorotic lesions later turns to necrotic lesions or yellow spots coalesce to form mosaic pattern and develops concentric rings on mature leaves and fruits^12^. Basically, infection may be caused by a single virus or mixed infection by more than one virus together by forming disease complex^7^. In general, dynamics of mixed infection with two or more unrelated viruses are common in nature^16^. Indeed, mixed infection of CMV with *Potato virus Y*^17^, CMV with Pepper mottle virus^16,18^, CMV with Turnip yellow mosaic virus^19^, CMV with Watermelon mosaic virus^20^, CMV with Tomato leaf curl New Delhi virus^21^ and Cucurbit yellow stunting disorder virus with Zucchini yellow mosaic virus^22^ have been reported previously. The mixed infection allows diverse virus interactions and maintenance of viral genetic diversity. Symptoms induced by mixed viral infections were more severe than the single viral infection^16,23^. Besides, mixed infections tend to affect virulence, accumulation, symptomology and host range which ultimately results in unpredictable pathological consequences^24,25^. Moreover, virus interaction in host plant hews the evolutionary dynamic of viral populations^26^. Unraveling the viruses concerned with mixed infections is vital for better understanding of the ecology and evolution of viral diseases which are essential for viral disease prevention and formulating management strategies^25^. The prime objective of the present study is to explore the mixed infections of CMV with tospoviruses in chilli under natural field conditions of Tamil Nadu in India.

## Results

### Identification of viruses in the infected plants

**Figure 1:**
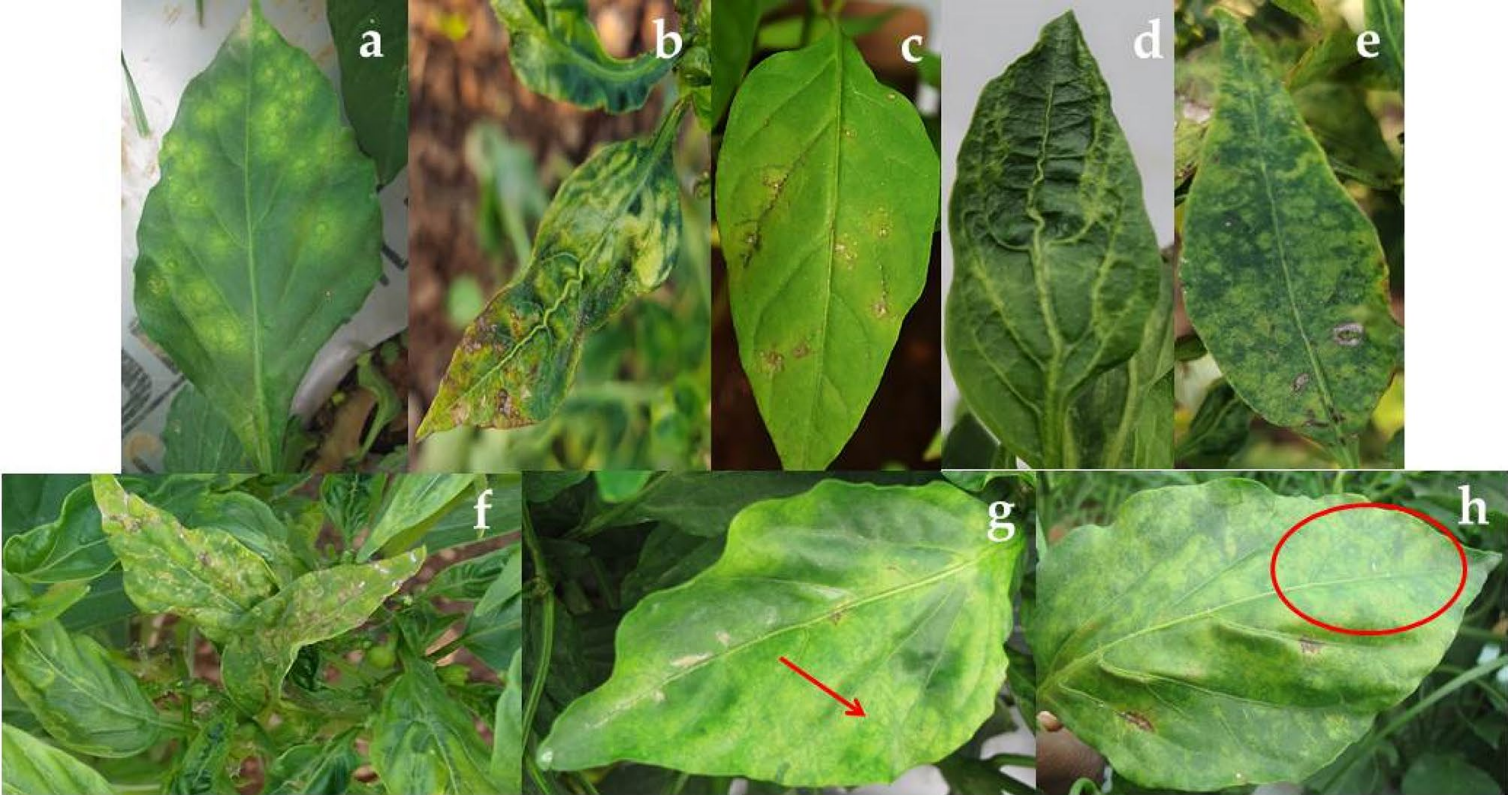
Symptoms observed on infected chilli plants: (**a**) cocncentric chlorotic ring spots; (**b**) necrotic lesions; (**c**) veinal necrosis; (**d**) leaf malformation; (**e**, **f**) necrosis along with mosaic (recorded mixed infection of CMV and GBNV); (**g**, **h**) mosaic with chlorotic ring spots; mark indicates concentric chlorotic rings (recorded mixed infection of CMV and CaCV).

**Figure 2:**
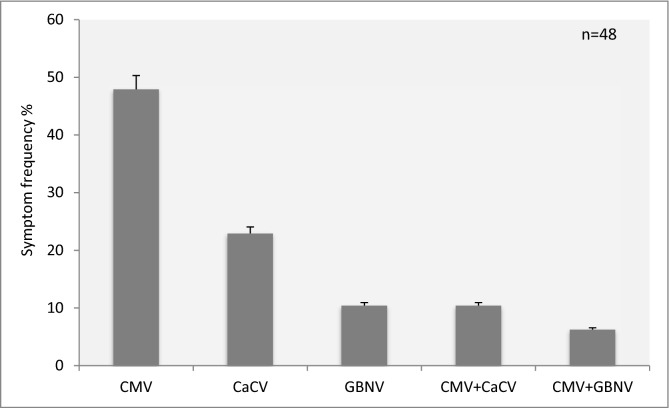
Per cent disease incidences of CMV, CaCV and GBNV infections of chilli in Tamil Nadu. The bar graph indicates that infection ratios of CMV (47.91%), GBNV (22.91%), CaCV (10.41%), CMV with CaCV (10.41%) and CMV with GBNV (6.25%) in chilli.

A total of 26 chilli samples of severe mosaic, mosaic mottling, leaf malformation and filiformity; 13 samples with concentric chlorotic ring spots and 9 samples with veinal necrosis and necrotic lesions were collected (Fig. 1). Of these, CMV was detected in 23 samples (47.91%); CaCV was recorded in 11 samples (22.91%) and GBNV in 5 samples (10.41%). The co-infection of CMV with CaCV was detected in 5 samples (10.41%) and CMV with GBNV was recorded in 3 samples (6.25%) (Fig. 2). The symptomatic leaf samples of mosaic mottling along with concentric chlorotic ring spots and mosaic with veinal necrosis were suspected for mixed infection based on symptomology. The symptomatic leaf samples were subjected to RT-PCR analysis with CMV coat protein gene specific primer and tospovirus degenerate primer pairs. The coat protein gene fragment of CMV and replicase protein (RdRP) fragment of tospovirus were amplified with an amplicon size of around 657 bp and 850 bp, respectively (Supplementary Fig. 1). The amplification was observed for CMV and tospovirus as mixed infection as well as for single infections. The respective amplicons (mixed infection) were cloned and sequenced. Sequence analysis explicates that tospoviruses associated with CMV as Groundnut bud necrosis virus and Capsicum chlorosis virus.

### Symptomatological study

**Figure 3:**
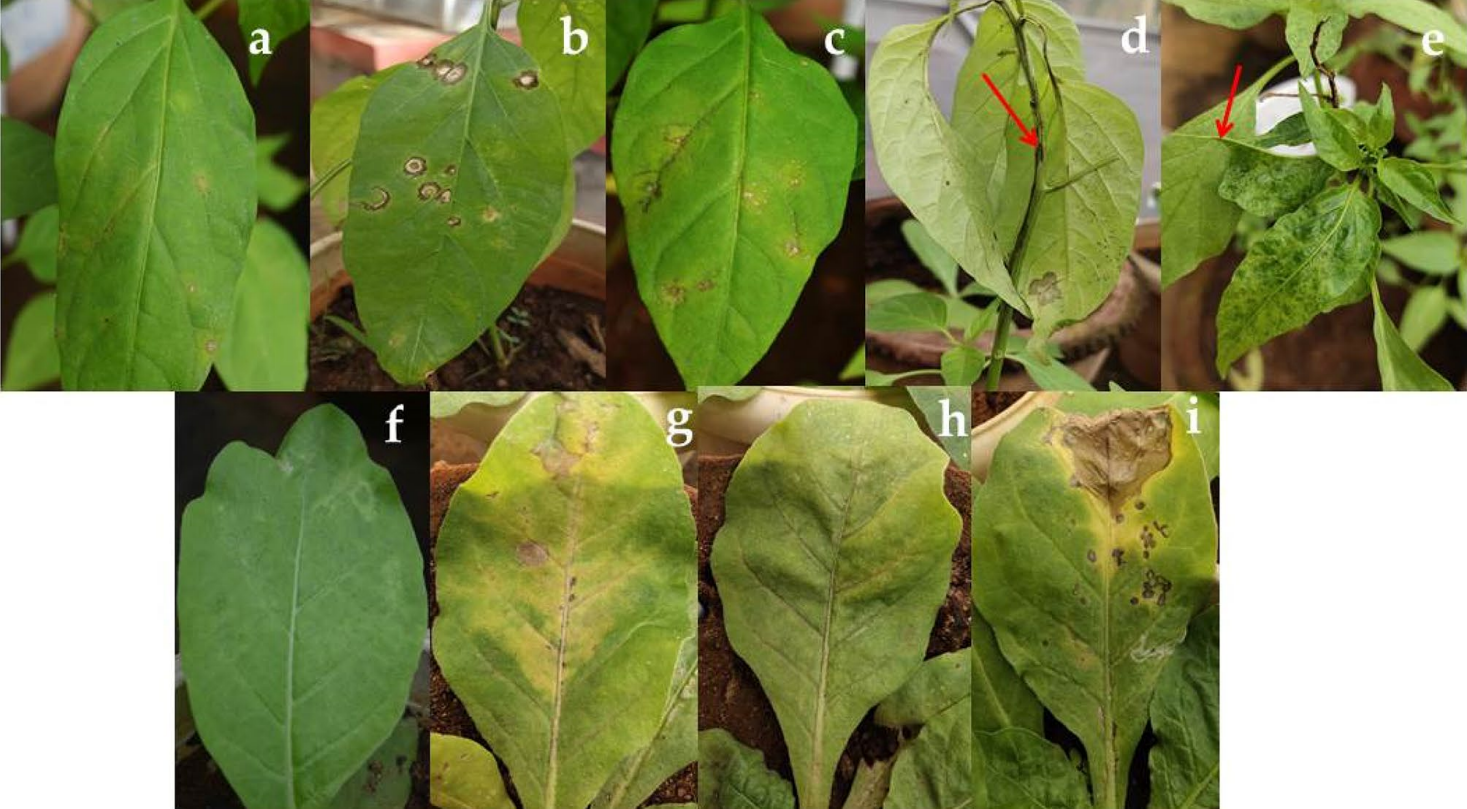
Symptomatological studies on sap inoculated chilli and *Nicotinana* plants for mixed infection: (**a**) chilli plant showing chlorotic lesions at 2 dpi (mixed infection of CMV with CaCV); (**b**) chilli plant showing necrotic lesions at 3–4 dpi (mixed infection of CMV with CaCV); (**c**) veinal necrosis on chilli at 5 dpi (mixed infection of CMV with GBNV); (**d**) stem necrosis on chilli at 7 dpi (mixed infection of CMV with GBNV); (**e**) chilli plant showing leaf filiformitity and mosaic mottling at 10–12 dpi (mixed infection of CMV with CaCV); (**f**) chlorotic lesions on *Nicotiana* at 2–3 dpi; (**g**) mosaic and necrotic lesions on *Nicotiana* at 7–8 dpi (mixed infection of CMV with CaCV); (**h**, **i**) mosaic and Necrotic lesions on *Nicotiana* at 7 dpi (mixed infection of CMV with GBNV)*.*

Symptomatic leaf samples (confirmed for mixed infection) were sap inoculated onto chilli and *Nicotiana* host plants to study the symptomatology and transmission nature. Sap inoculated *Nicotiana* and chilli host plants produced symptoms after 2–3 days of post inoculation (dpi). Initially, localized chlorotic lesions were observed on inoculated leaves of chilli plant at 2 dpi (Fig. 3a) which turned to necrotic lesions at 3–4 dpi (Fig. 3b). After 5 dpi, it exhibited veinal necrosis (Fig. 3c) followed by stem necrosis symptom (7 dpi) on inoculated plants (Fig. 3d). In contemplate, the symptomatic leaves started to wither after 9 dpi and the newly emerging leaves were observed to be having the systemic symptom of mosaic mottling followed by leaf filiformity after 10–12 dpi (Fig. 3e). The sap inoculated *Nicotiana* plants produced localized chlorotic lesions on 2–3 dpi (Fig. 3f) which turned into necrosis at 5 dpi (Fig. 3g). After 7 dpi, the systemic mosaic and necrosis were observed on newly emerged leaves (Fig. 3h,i). Furthermore, the co-existence of CMV with tospovirus in the inoculated plant samples was confirmed by RT-PCR using nucleocapsid gene specific primers of CaCV (Supplementary Fig. 2a) and GBNV (Supplementary Fig. 2b). Indeed, inoculated plant samples after 12 dpi were positively amplified for both CMV and tospovirus.

### Structural characterization

**Figure 4:**
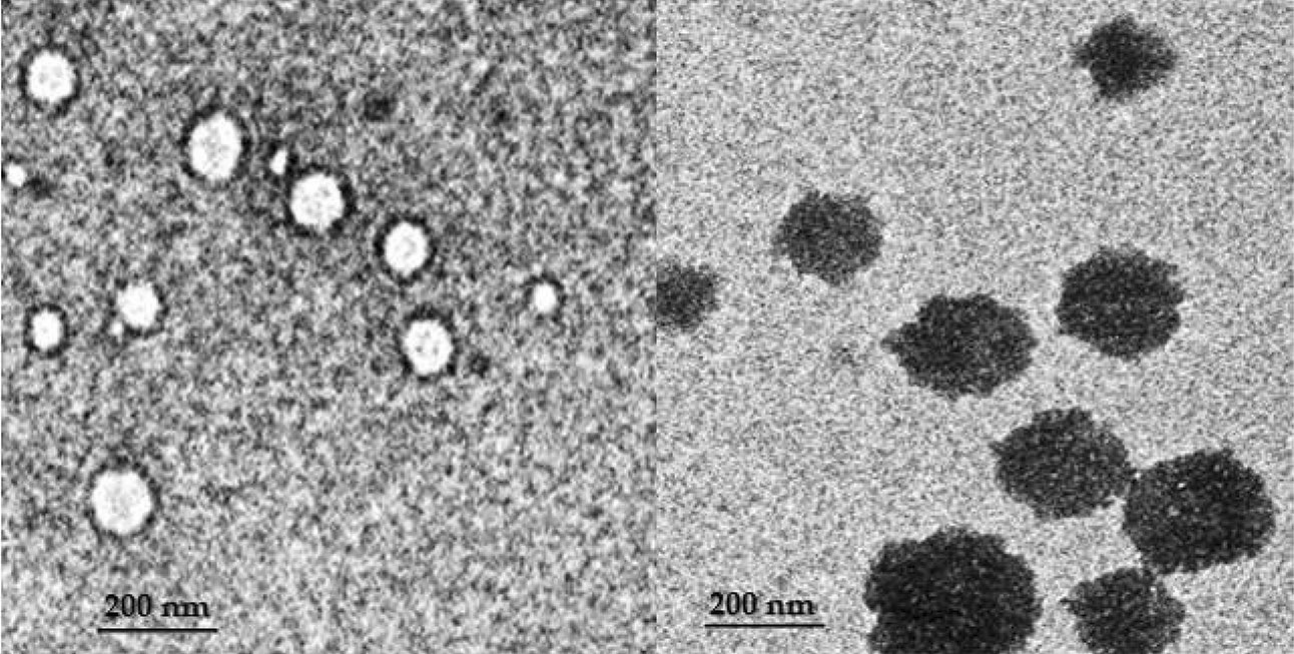
Electron micrograph of PDA stained 24–72 nm diameter of isometric CMV particles (right) and 80–120 nm diameter of quasi spherical tospovirus particles in the infected chilli plant sample.

Inoculated plant samples were partially purified and observed under transmission electron microscope to determine the particle morphology of viruses associated with mixed infection. Electron micrograph of infected plants showed isometric virus particles of 24–52 nm in diameter and quasi spherical virus particles of 70–120 nm in the sample (Fig. 4). From the results, isometric particles identified as CMV and spherical particles identified as tospoviruses. Thus, evinces the co-existence of CMV and tospovirus in the infected chilli plants.

### Molecular characterization and sequence analysis

**Figure 5:**
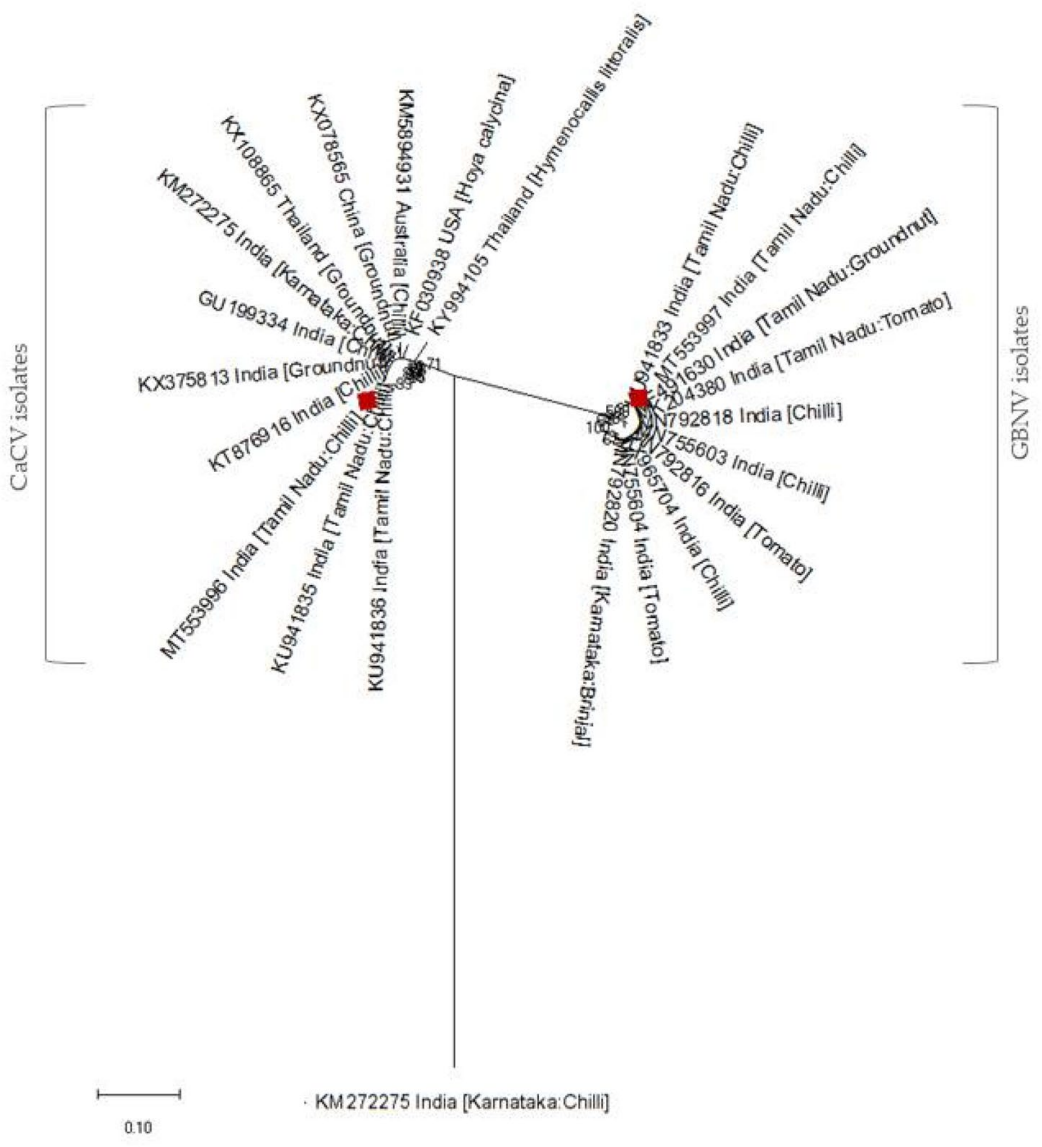
Phylogenetic relationship of TN CaCV and TN GBNV isolates with other isolates based on RdRp segment. Phylogenetic tree generated using MEGA 7 software using Neighbor joining tree method with 1000 replicates and CMV (KM272278) isolate used as out group.

**Figure 6:**
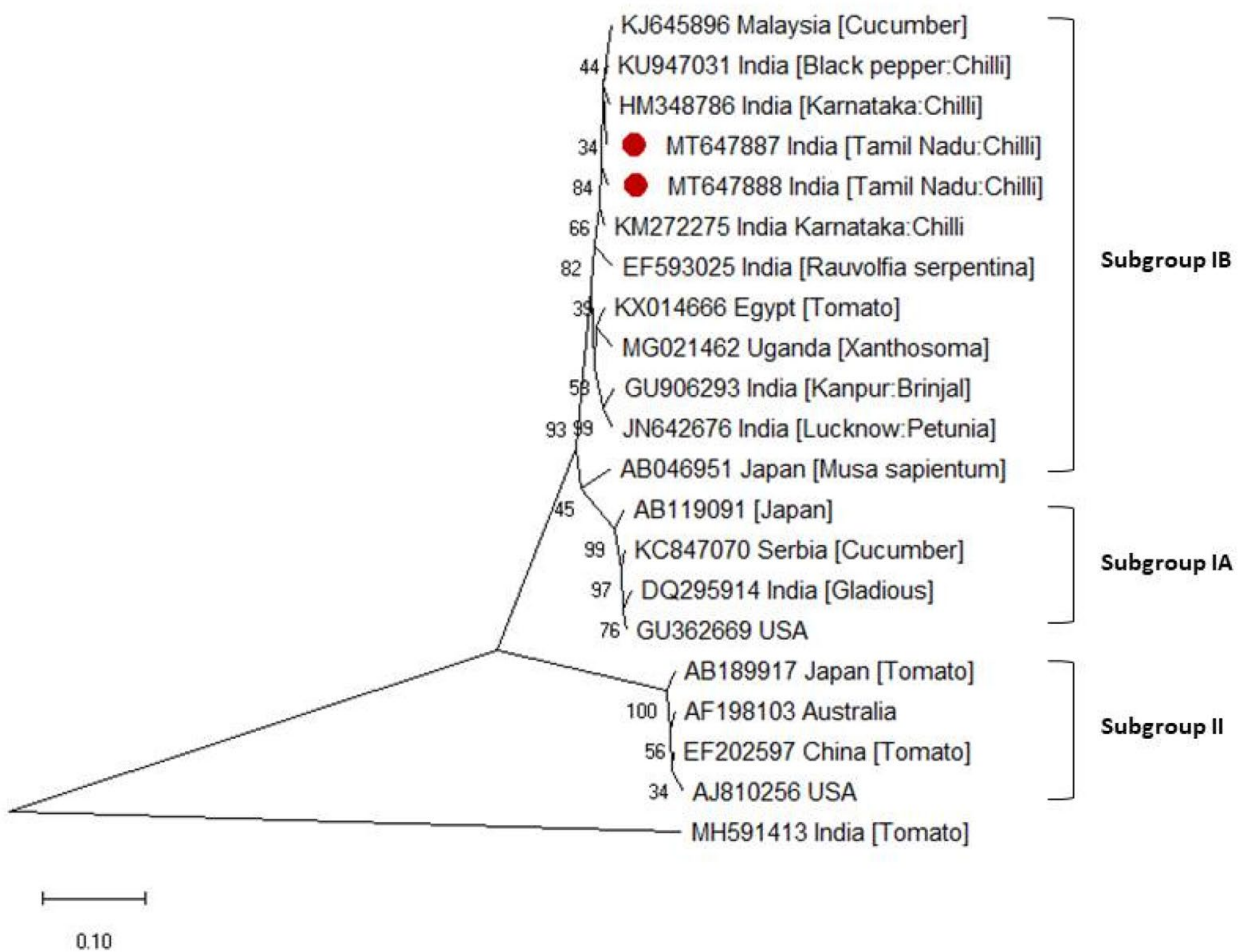
Phylogenetic relationship of CMV isolates of Tamil Nadu with other CMV isolates reported worldwide based on coat protein gene. Phylogenetic tree generated using MEGA 7 software using Neighbor joining tree method with 1000 replicates and GBNV (MH591413) isolate used as out group.

The nucleotide sequences of GBNV (MT553997) showed 98.96% of identity with chilli isolate of Trichy (KU941833) followed by groundnut isolate of Coimbatore (MF491630). Similarly, CaCV (MT553996) had an identity of 97.5% at nucleotide level with chilli isolate of Coimbatore (KU941835) followed by groundnut isolate of China (KX078565). Likewise, CMV (MT647887 and MT647888) isolates showed 97.5–98% identity with chilli isolate of Karnataka (HM348786) followed by 97.2–97.5% of identity with coat protein gene of chilli isolate (KM272275). Genetic divergence of TN CaCV and GBNV isolates have been evaluated through phylogenetic analysis of TN isolates with other member isolates inferred using neighbor joining tree method with 1000 replicates. Thus, phylogenetic analysis depicts diversified cluster formation of CaCV and GBNV isolates based on RdRp gene segment with other member GBNV and CaCV isolates of India (Fig. 5). Correspondingly, CMV isolate (MT647887) clustered with Indian CMV isolates and other members of subgroup IB (Fig. 6). Thus implies the present Cucumber mosaic virus isolate belongs to subgroup IB. From, sequence analysis complete coat protein gene of CMV was determined to be 657 nucleotides encodes for 218 amino acids. Coat protein gene sequence analysis of CMV isolates explicates unique amino acid substitutions when compared with CMV strain causing single infection. Comparative amino acid sequence alignment showed amino acid proline (Pro^129^) substituted with serine (Ser^129^) at 129^th^ position in coat protein of CMV (Supplementary Fig. 3). Inevitably, CMV isolates recorded to cause co-infection with tospoviurses possess unique amino acid substitution of Ser^129^ over Pro^129^ in the coat protein. Similarly, amino acid substitution of threonine over serine (Ser^137^–Thr^137^) observed in coat protein comparatively with CMV isolate causing single infection.

## Discussions

Several epidemiological studies depicts that single host plant affected by more than one virus implies the occurrence of mixed virus infection. In general, mixed viral infections are more common^21^. Tospoviruses (CaCV and GBNV) infected chilli plants showed concentric rings on mature leaves and chlorotic spots on younger leaves^12^. Indeed, CMV infected chilli plants showed mosaic mottling, puckering, shoe string or filiformity and stunted growth^26,27^. Based on symptomology, symptomatic leaf samples of mosaic mottling along with concentric chlorotic ring spots and mosaic with veinal necrosis were suspected for mixed infection. Since, symptomology based detection would not be reliable and it has to be further confirmed and validated either by serological or by molecular method of detection^12^. RT-PCR based method will be the reliable to underpin the mixed infection and to index the associated viruses. It is more accurate to detect the viruses even at very low titer in the infected leaf samples than serology based assays^28^. From the analysis, it is clearly demonstrated that the tospoviruses associated with CMV were identified as Groundnut bud necrosis virus and Capsicum chlorosis virus. The symptomatological study depicts that GBNV and CaCV produces similar symptoms on chilli plant and it is very difficult to distinguish them based on symptomology. The sap inoculation of tospovirus (GBNV and CaCV) chilli isolate on *Nicotiana* and cowpea host plants produced localized chlorotic lesions followed by necrotic lesions at 4–5 dpi^12,29^. The CaCV certainly induces chlorotic spots initially and later developed necrosis around the central spot^8^. Intrinsically, mechanically inoculated plant samples after 12 dpi were positively amplified for both CMV and tospovirus in RT-PCR.

Electron microscopic visualization and evaluation helps to study the particle morphology for detection of mixed viral infections. Substantially, diagnosis of viral diseases by TEM utters rapid and accurate identification towards mixed infection^30^. Hence, electron microscopic examination of infected plant samples corroborated the co-existence of isometric cored particle of CMV with quasi spherical particle of tospovirus. The results have been substantiated with previous findings^13,31–33^.

The nucleotide sequences of coat protein of CMV indicates that CMV isolate belong to subgroup IB. Based on serology and nucleic acid hybridization, CMV isolates are classified into subgroup I and II and further CMV subgroup I divided into IA and IB based on coat protein gene sequence and phylogenetic analysis^34^. Coat protein of CMV comprised of 657 nucleotides putatively translated into 219 amino acids. CMV can be categorized as chlorosis inducing strain (Ser^129^ or Leu^129^) and mosaic inducing strain (Pro^129^) based on the position of 129^th^ amino acid of coat protein gene^35^. Certainly, amino acid 129 of coat protein is a genetic determinant responsible for symptom induction in host plant and in certain cases it act as virulence determinant^36^. Amino acid substitution of Ser^129^ over conserved Pro^129^ induces chlorosis in the host rather necrosis by disrupting the functions of chloroplast^37^. More likely, cholorosis induction in the infected plant determined by structural alteration in coat protein of CMV incited by conserved (pro^129^) amino acid substitution^38^. Comparative amino acid sequence alignment vindicated CMV isolates associated with mixed infection is a chlorosis inducing strain (substituted with Ser^129^). Whereas, CMV causing single infection are prevalently mosaic inducing strains (possess Pro^129^). Increased flexibility of βE-αEF loop (129–136 amino acids) of coat protein has direct correlation with pathogenesis of virus. Moreover, flexibility of βE-αEF loop is regulated by 129^th^ amino acid properties^39^. In general, frequent reassortment and recombination occurs in segmented viruses over monopartite viruses^40^. Amicably, CMV undergoes prompt of genetic changes through reasssortment and recombination^27,41,42^. Thus, evolutionary mechanism is a characteristic attribution of multipartite viruses which plays significant role in emergence and interspecies transmission of viruses^43^. Any single variation in amino acid or nucleotides may effect on stability and infectivity of virus. Hence, thus apparent variation observed on multipartite TN CMV isolate suspected to be associated with co-adoption or co-existence of CMV with other viruses. Over all the accumulating evidences suggests that amino acid 129 (chlorosis induction) besides symptom determinant may also have correlation with co-existence of CMV with tospoviruses.

Mixed virus infections will intensify the disease dynamic in host plants comparatively than single virus infection. In some cases, symptom induced by one virus can be masked by other virus in combined infections. Mixed infection in host plants will results in maintaining the genetic diversity of viruses. Thus may inflict to the emergence of novel genetic phenotypes or strains of virus. It is very crucial to study the significant role of mixed infection in altering genetic diversity of viral population. In the present study, we explicated the co-existence of Cucumber mosaic virus with Capsicum chlorosis virus and Groundnut bud necrosis virus in chilli under natural field conditions of Tamil Nadu in India. Indeed, CMV, GBNV and CaCV are the most notorious and economically important viruses reported to be causing mosaic and necrosis on most of vegetable crops. Hence, combined infection of these destructive viruses may menace to chilli cultivation. Moreover, frequency of occurrence of mixed infection of mosaic and necrosis disease in chlli becoming increasingly diverse and alarming currently in chilli growing areas of Tamil Nadu. To our knowledge, this is the first report for combined infection of cucumo- and tospoviruses in chilli. This preliminary study sought to help in further future studies on combined infections of CMV with other plant viruses. Although, the mechanism behind the interaction of viruses between different groups on a single host during mixed infections is yet to be studied in detail.

## Materials and methods

### Field survey and symptomatological study

A systemic field survey was conducted during 2018–2019 in Coimbatore, Thiruvannamalai, Dindigul, Tirunelveli, Namakkal, Salem, Krishnagiri and Theni districts of Tamil Nadu in India. Plants showing characteristic virus disease symptoms were identified and the symptomatic leaf samples with mosaic, veinal necrosis, chlorotic rings, necrotic ring spots, mosaic mottling, leaf malformation and stunted growth were collected. Infected chilli samples (observed for mixed infection) with 0.1 M phosphate buffer (pH 0.7) were mechanically inoculated on chilli and *Nicotiana benthamiana* plants at 2–3 leaf stage by gentle aberration on carborundum dusted leaves^43^. The inoculated plants were maintained at 30 ± 2 °C in greenhouse of Department of Plant Pathology, Tamil Nadu Agricultural University, Coimbatore (India) under insect proof cage for the symptom expression.

### Partial purification of virus and electron microscopy

The virus from infected plants was purified as per methodology^44^. 250 g of infected sample ground using liquid nitrogen and centrifuged at higher speed. The obtained pellet suspended in 200 µl of storage buffer containing 5 mM sodium borate and 0.5 mM EDTA. Briefly, 10 µl of 0.01% of BSA was loaded into 400 mesh copper grid and draw off from grid after few seconds. Subsequently, 2 µl of purified virus sample coated into grid kept for 3–5 min and then, washed thrice with sterile water. Consequently, virus particles have been stained with 2% phospho tungstic acid. The excess stain has been wiped off from edges of grid with filter paper and allowed for air dry. Eventually, grid was observed under TEM with EDAX (FEI, Technai) at 70,000–90,000 magnification and documented.

### RNA extraction and RT-PCR analysis

Total RNA was extracted from the collected symptomatic leaf samples using TRIzol reagent^45^. The first stand complementary DNA (cDNA) was synthesized from the extracted RNA to detect the viruses associated with infections. For the cDNA synthesis of CMV, 20 µl reaction mixture (9 µl- sterile water; 4 µl- 5× reaction buffer; 2 µl- dNTPs; 1 µl- random primer; 1 µl- reverse transcriptase; 1 µl- RNase inhibitor; 1 µg-total RNA) was used (RevertAid, Fermentas, India) and incubated at 42 °C for 60 min followed by 70 °C for 5 min^46^. For tospovirus, the reaction mixture contains total RNA, random primer and nuclease free water up to 12.5 µl were incubated at 65 °C for 5 min followed by 5× reaction buffer, 10 mM dNTP mix, RNase inhibitor and reverse transcriptase were added and incubated for 1 h at 42 °C^47^. The presence of CMV was confirmed through RT-PCR with CMV coat protein gene specific primer (RsCMV-F and RsCMV-R) with a cyclic condition of 94 °C for 2 min followed by 94 °C for 30 s of denaturation, 59 °C for 30 s of annealing, 72 °C for 60 s with final extension of 72 °C for 10 min^48^. The tospovirus was confirmed through RT-PCR using tospovirus universal primer (gL3637 and gL4435C) corresponding to L segment (RdRp) with the cycling condition of 94 °C for 5 min followed by 35 cycles of 94 °C for 30 s, 56 °C for 1 min and 72 °C for 1 min with 72 °C for 10 min of final extension^44,49^.

### Cloning and sequence analysis

The positive amplicons of CMV (coat protein) and tospoviruses (replicase protein) were purified using GenJET PCR purification kit (Themo scientific Inc.) and cloned into pGEM-T easy vector (Promega). Two independent clones were sequenced at both orientations with M/s Barcode Biosciences, Bangalore. Data base searches performed with NCBI BLAST (http://blast.ncbi.nlm.nih.gov) and comparative amino acid and nucleotide sequence analysis carried out using Clustal W (www.ebi.ac.uk). Sequences were aligned using Bio-Edit Sequence Editor program 7.2. The phylogenetic relationships among the isolates were analyzed using MEGA 7.0 software (www.megasoftware.net) by Neighbor joining tree method with 1000 bootstrap replication^12,50^.

## Supplementary information

Supplementary Informations.
