# Supplementary material for: Co-existence of chlorosis inducing strain of Cucumber mosaic virus with tospoviruses on hot pepper (Capsicum annuum) in India
Source: Sci Rep. 2021 Apr 22;11:8796. doi: 10.1038/s41598-021-88282-9 (PMC8062535; doi:10.1038/s41598-021-88282-9)
Supplement: Supplementary file 1 — Supplementary Informations. [file 41598_2021_88282_MOESM1_ESM.docx]

**Co-existence of chlorosis inducing strain of *Cucumber mosaic virus* with tospoviruses on hot pepper (*Capsicum annuum*) in India**

Vinodhini, J., Rajendran, L., Abirami, R and Karthikeyan, G*

Department of Plant Pathology,

Tamil Nadu Agricultural University, Coimbatore - 641 003, Tamil Nadu, India

* Corresponding author ([agrikarthi2003@gmail.com](mailto:agrikarthi2003@gmail.com))

**Supplementary Table1** Details on CMV, CaCV and GBNV isolates and their GenBank accession numbers used in the study

| **S.no** | **Accession number** | **Host** | **Country** |
| --- | --- | --- | --- |
| **Cucumber mosaic virus (CMV)** | | | |
| 1 | MT647887  (Present study) | *Capsicum annuum* | India |
| 2 | MT647888  (Present study) | *Capsicum annuum* | India |
| 3 | MT395346 | *Capsicum annuum* | India |
| 4 | MT396263 | *Capsicum annuum* | India |
| 5 | HM348786 | *Capsicum annuum* | India |
| 6 | KM272275 | *Capsicum annuum* | India |
| 7 | KU947031 | *Piper nigrum* | India |
| 8 | KJ874248 | *Trichosanthes cucumerina* | India |
| 9 | JN054635 | *Cucumis sativus* | Malaysia |
| 10 | KX014666 | *Lycopersicon esculentum* | Egypt |
| 11 | GU111229 | *Lycopersicon esculentum* | India |
| 12 | Y18137 | *Lycopersicon esculentum* | France |
| 13 | AJ276481 | *Melandrium firmum* | Korea |
| 14 | D10538 | *Cucumis melo* | USA |
| 15 | AJ831578 | *Lilium longiflorum* | India |
| 16 | M21464 | *Capsicum annuum* | Australia |
| 17 | EU665002 | *Tagetes erecta* | China |
| 18 | AB176847 | *Cucumis sativus* | Japan |
| 19 | AF127976 | *Lactuca saligna* | USA |
| **Capsicum chlorosis virus (CaCV)** | | | |
| 20 | MT553996  (Present study) | *Capsicum annuum* | India |
| 21 | KU941835 | *Capsicum annuum* | India |
| 22 | KX078565 | *Arachis hypogaea* | China |
| 23 | KU941836 | *Capsicum annuum* | India |
| 24 | KX375813 | *Groundnut* | India |
| 25 | KM589493 | *Capsicum annuum* | Australia |
| 26 | KX108865 | *Arachis hypogaea* | Thailand |
| 27 | KY994105 | *Hymenocallis littoralis* | Thailand |
| 28 | GU199334 | *Capsicum annuum* | India |
| 29 | KT876916 | *Capsicum annuum* | India |
| **Groundnut bud necrosis virus (GBNV)** | | | |
| 30 | MT553997  (Present study) | *Capsicum annuum* | India |
| 31 | KU941833 | *Capsicum annuum* | India |
| 32 | MF491630 | *Arachis hypogaea* | India |
| 33 | MF498871 | *Arachis hypogaea* | India |
| 34 | MN792818 | *Capsicum annuum* | India |
| 35 | MN792820 | *Solanum melongena* | India |
| 36 | MH754141 | *Lycopersicon esculentum* | India |
| 37 | MN755603 | *Capsicum annuum* | India |
| 38 | KY940037 | *Solanum torvum* | India |
| 39 | KX965704 | *Capsicum annuum* | India |

**Co-existence of chlorosis inducing strain of *Cucumber mosaic virus* with tospoviruses on hot pepper (*Capsicum annuum*) in India**

Vinodhini, J., Rajendran, L., Abirami, R and Karthikeyan, G*

Department of Plant Pathology,

Tamil Nadu Agricultural University, Coimbatore - 641 003, Tamil Nadu, India

* Corresponding author ([agrikarthi2003@gmail.com](mailto:agrikarthi2003@gmail.com))

**
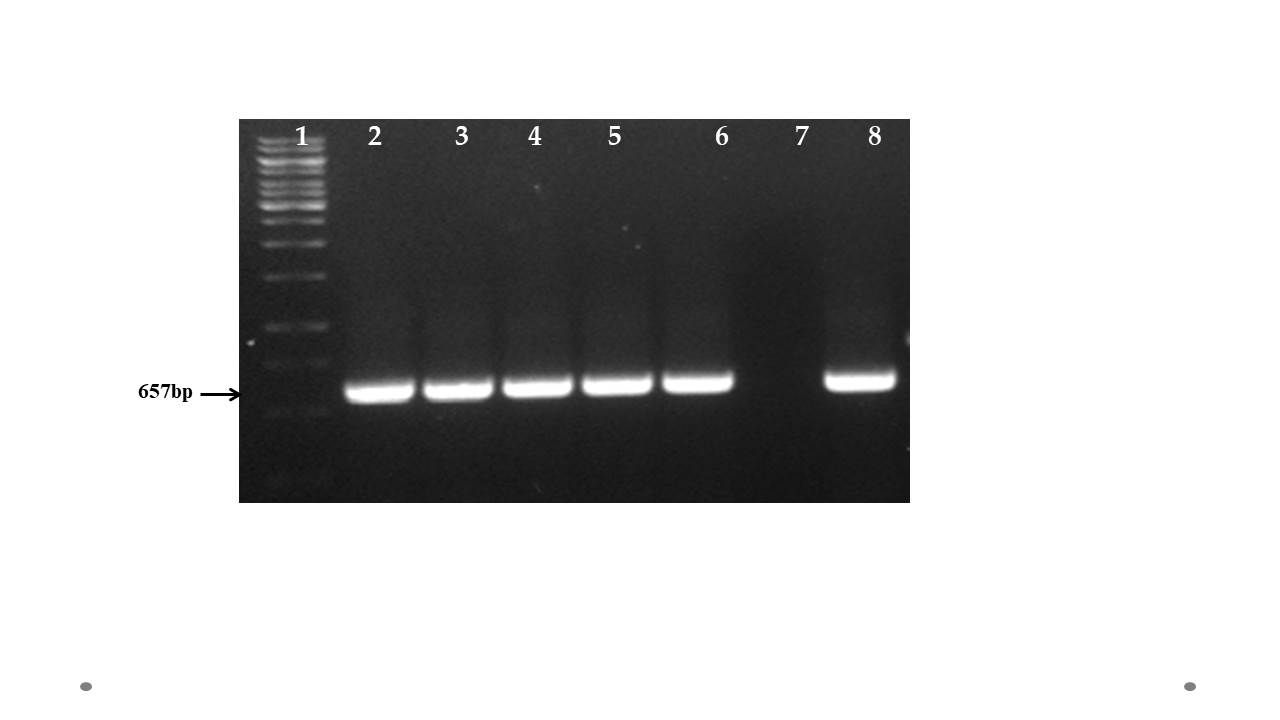
**

1- 1kb ladder;2- Namakkal sample; 3- Salem sample; 4- Krishnagiri sample; 5- Dindigul sample; 6- Tirunelveli sample; 7- Positive control; 8 – Negative control (CMV infected tomato)

**Supplementary Fig 1a RT-PCR analysis of test plants using CMV CP gene specific primers (RsCMV-F 5’ATGGACAAATCTGAATCAAC3’; RsCMV-R 5’TCAAACTGGGAGC ACCC3’)**


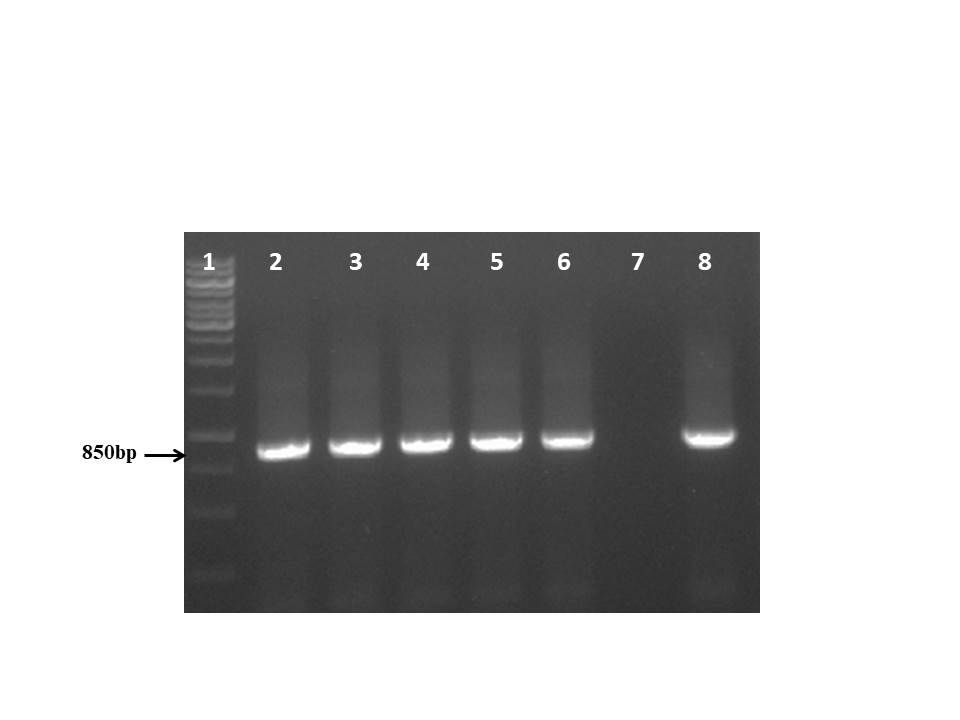


1- 1kb ladder;2- Namakkal sample1 (CMV+CaCV); 3- Namakkal sample2 (CMV+GBNV); 4- Krishnagiri sample (CMV+CaCV); 5- Dindigul sample (CMV+CaCV); 6- Tirunelveli sample (CMV+GBNV); 7- Negative control; 8 –Positive control (GBNV infected tomato)

**Supplementary Fig 1b RT-PCR analysis of test plants using tospovirus gL primers (gL3637/F 5’CCTTTAACAGTDGAAACAT3’; gL4435/R-5’CATDGCRCAAGARTGRTARA CAGAC3’)**

**Co-existence of chlorosis inducing strain of *Cucumber mosaic virus* with tospoviruses on hot pepper (*Capsicum annuum*) in India**

Vinodhini, J., Rajendran, L., Abirami, R and Karthikeyan, G*

Department of Plant Pathology,

Tamil Nadu Agricultural University, Coimbatore - 641 003, Tamil Nadu, India

* Corresponding author ([agrikarthi2003@gmail.com](mailto:agrikarthi2003@gmail.com))

**
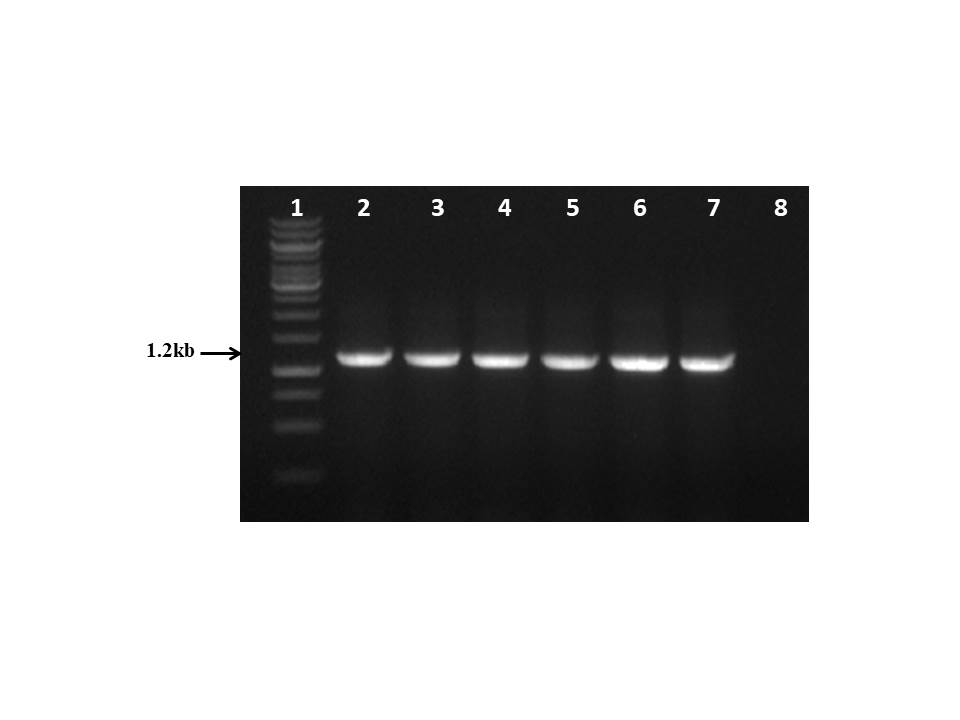
**

1- 1kb ladder;2- Namakkal sample1 (CMV+CaCV); 3- Namakkal sample2 (CaCV); 4- Krishnagiri sample (CMV+CaCV); 5- Krishnagiri sample (CaCV); 6- Dindigul sample (CMV+CaCV); 7- Positive control (CaCV infected chilli); 8 – Negative control

**Supplementary Fig 2a RT-PCR analysis of test plants using CaCV nucleocapsid gene specific primers (GKCaCVCPF1: 5’AACCAATAGTTTGCCTCCG3’; GK CaCVCPR1: AGAGCAATCGAGGCACTA)**

**
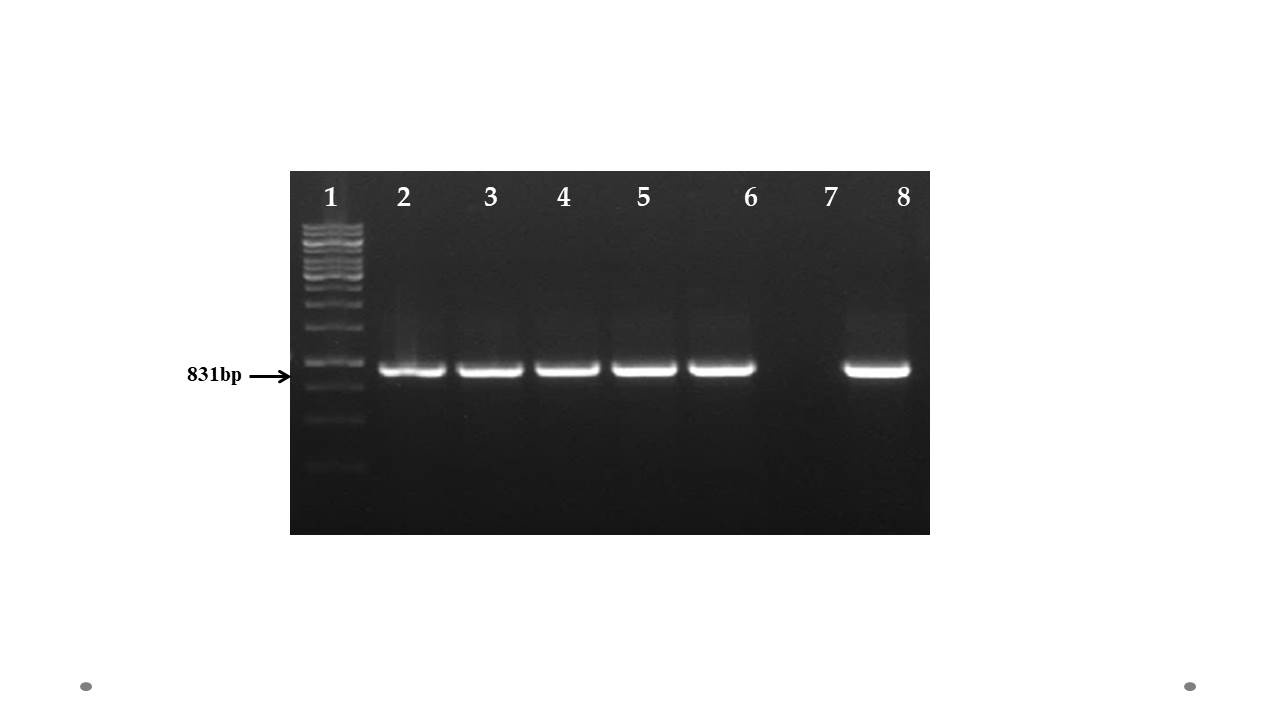
**

1- 1kb ladder;2- Namakkal sample1 (CMV+GBNV); 3- Namakkal sample2 (CMV+GBNV); 4- Dindigul sample (CMV+CaCV); 5- Dindigul sample2 (CMV+CaCV); 6- Tirunelveli sample (CMV+GBNV); 7- Negative control; 8 –Positive control (GBNV infected tomato)

**Supplementary Fig 2b RT-PCR analysis of test plants using GBNV nucleocapsid gene primers (GKPBNVCP F-5’ATGTCTAACGTYAAGCAGCTC3’; GKPBNVCP R- 5’TTACAATTCCAGCGAAGGAC3’)**

**Co-existence of chlorosis inducing strain of *Cucumber mosaic virus* with tospoviruses on hot pepper (*Capsicum annuum*) in India**

Vinodhini, J., Rajendran, L., Abirami, R and Karthikeyan, G*

Department of Plant Pathology,

Tamil Nadu Agricultural University, Coimbatore - 641 003, Tamil Nadu, India

* Corresponding author ([agrikarthi2003@gmail.com](mailto:agrikarthi2003@gmail.com))

**
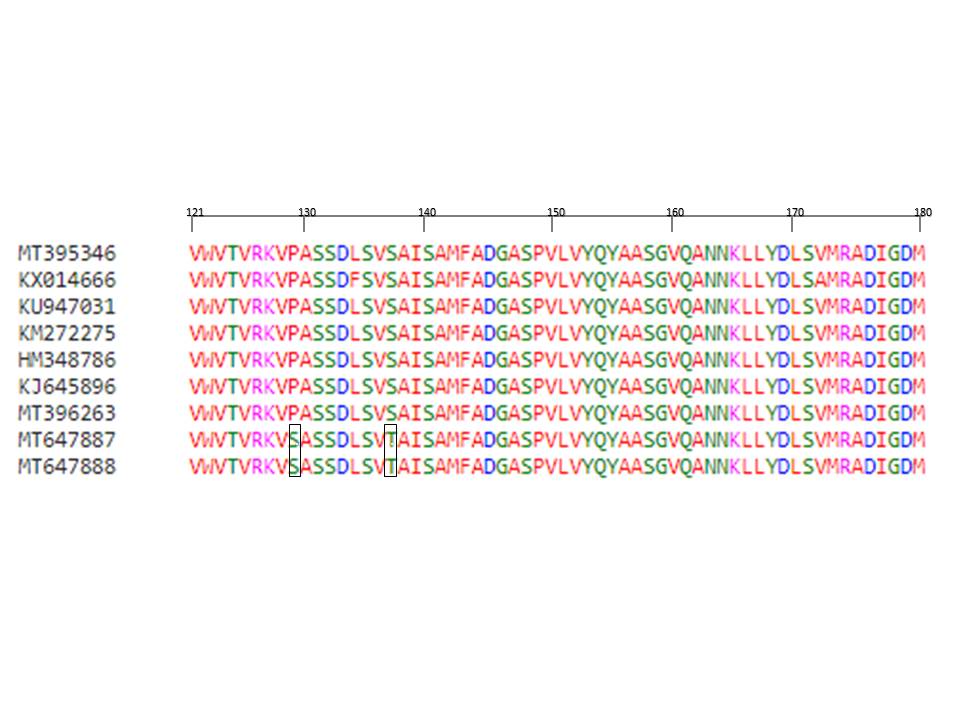
**

**Supplementary Fig 3 Comparative amino acid sequence alignment illustrates amino acid proline substituted by serine at 129 position of CP of CMV. Similarly, amino acid serine substituted by threonine at 137 positions (MT647887 and MT647888 are coat protein gene of CMV involved in mixed infection with tospovirus).**
